# Systematic Review and Meta-analysis of Genetically Informed Research: Associations Between Parent Anxiety and Offspring Internalizing Problems

**DOI:** 10.1016/j.jaac.2020.12.037

**Published:** 2021-07

**Authors:** Yasmin I. Ahmadzadeh, Tabea Schoeler, Meredith Han, Jean-Baptiste Pingault, Cathy Creswell, Tom A. McAdams

**Affiliations:** a King’s College London, United Kingdom; b University of Oslo, Norway; c University College London, United Kingdom; d University of Oxford, United Kingdom

**Keywords:** genetics, meta-analysis, offspring internalizing, parent anxiety, quasi-experimental

## Abstract

**Objective:**

Parent anxiety is associated with offspring internalizing problems (emotional problems related to anxiety and depression). This may reflect causal processes, whereby exposure to parent anxiety directly influences offspring internalizing (and/or vice versa). However, parent–offspring associations could also be attributable to their genetic relatedness. A systematic review and meta-analysis were conducted to investigate whether exposure to parent anxiety is associated with offspring internalizing after controlling for genetic relatedness.

**Method:**

A literature search across 5 databases identified 429 unique records. Publications were retained if they used a quasi-experimental design in a general population sample to control for participant relatedness in associations between parent anxiety and offspring internalizing outcomes. Publications were excluded if they involved an experimental exposure or intervention. Studies of prenatal and postnatal anxiety exposure were meta-analyzed separately. Pearson’s correlation coefficient estimates (*r*) were pooled using multilevel random-effects models.

**Results:**

Eight publications were retained. Data were drawn from 4 population cohorts, each unique to a quasi-experimental design: adoption, sibling-comparison, children-of-twins or in vitro fertilization. Cohorts were located in northern Europe or America. Families were predominantly of European ancestry. Three publications (N_families_ >11,700; offspring age range, 0.5–10 years) showed no association between prenatal anxiety exposure and offspring internalizing outcomes after accounting for participant relatedness (*r* = .04; 95% CI: −.07, .14). Six publications (N_families_ >12,700; offspring age range, 0.75–22 years) showed a small but significant association between concurrent symptoms in parents and offspring after accounting for participant relatedness (*r* = .13; 95% CI: .04, .21).

**Conclusion:**

Initial literature, derived from homogeneous populations, suggests that prenatal anxiety exposure does not cause offspring internalizing outcomes. However, postnatal anxiety exposure may be causally associated with concurrent offspring internalizing via nongenetic pathways. Longitudinal stability, child-to-parent effects, and the role of moderators and methodological biases require attention.

Anxiety disorders are the most prevalent class of mental disorders worldwide.^1^ They are characterized by pervasive emotional and physical distress that can substantially restrict daily functioning. The median age of onset for anxiety disorders is 11 years.^2^ Anxiety symptoms and disorder diagnoses cluster within families, with disorder status among parents being a robust predictor of related internalizing problems among developing offspring.^3^, ^4^, ^5^ Internalizing problems encapsulate emotional symptoms characteristic of both anxiety and depression. Core internalizing symptoms include worry, fear, sadness, and withdrawal.^6^ Parent anxiety could pose an environmental risk for the development of offspring internalizing problems, for example, via modeling processes and social learning,^7^, ^8^, ^9^ or via fetal programming during pregnancy.^10^, ^11^, ^12^, ^13^ It is also possible that a child’s symptoms influence the parent’s symptoms, resulting in environmentally mediated transactional effects between parents and offspring.^14^, ^15^, ^16^ However, genetic transmission from parents to offspring is likely to at least partially account for symptom associations across generations. It is important to distinguish the potential environmental effects of familial exposure from associations attributable to genetic relatedness in families, to inform the development of successful intervention and prevention strategies.

Previous research on associations between parent anxiety and offspring internalizing outcomes has mostly relied on observational studies, where researchers may adjust for measured confounding but cannot account for unobserved variables, including genetics (eg, 17, 18, 19, 20). This is a major limitation that leads to ambiguous results, where potential causal pathways operating between parents and offspring (ie, if a parent’s symptoms directly influence the child’s symptoms and vice versa) are indistinguishable from influence by other common causes (eg, if the same genes influence symptoms in both parents and offspring). Population variance in anxiety and related internalizing problems is attributable in part to genetic influences,^21^, ^22^, ^23^ with the same genetic factors found to act in multiple internalizing phenotypes across the life span.^24^, ^25^, ^26^ It is therefore reasonable to expect that genetic factors influencing anxiety in adult parents may also act in their genetically related offspring, manifesting as similar problems during childhood. This results in passive gene–environment correlation, whereby the environment shared by parents and offspring is passively correlated with the genes that they share.^27^ Across time, it is possible for genetically influenced behaviors in parents and offspring to evoke changes in one another, as exemplified by longitudinal studies showing dynamic, transactional processes between family members.^14^, ^15^, ^16^ Together, existing evidence from genetic and longitudinal research suggests that purely observational studies cannot provide robust conclusions as to whether and how parent anxiety might directly influence the development of internalizing problems in a child. It is important to first control for confounding by shared genes and then to ask questions about the direction of effects between generations.

### Experimental Research

To explore the causal pathways linking parent anxiety and offspring internalizing, researchers can use either experimental or quasi-experimental methods. Experimental methods are used in medical research to test the effect of an exposure (or intervention) on an outcome, with randomized controlled trials typically labeled the gold standard. It is unfeasible and unethical to experimentally randomly assign children to be reared by parents who are experiencing anxiety symptoms vs patients who are not experiencing anxiety symptoms. Instead, researchers can temporarily exacerbate anxiety in parents or offspring, using controlled experiments, to examine whether increases in symptoms in one generation predict the same in the other. For example, parents’ fear responses to a novel toy can predict offspring fear and avoidance behaviors.^28^ Another experimental approach involves researchers treating parent or offspring symptoms and examining whether symptom improvements in one generation predict improvements in the other. Randomized controlled trial data show that treatment of anxiety in children is associated with a reduction in anxiety in parents.^29^^,^^30^ Treatment of anxiety in patients may also help to improve outcomes in children, in combination with child-focused treatment,^31^ although evidence is not always consistent.^32^^,^^33^ In sum, experimental research provides some evidence for causal effects between parent anxiety and offspring internalizing symptoms, but this research cannot inform on the nature of the association outside of the experimental setting or on the influence of genetic transmission in naturalistic settings. In the context of epidemiological research, experiments are limited because they tell us how things can be, rather than how they are in a population.

Quasi-experiments provide an alternative approach to experimental trials that can be used to test the possibility that causal mechanisms underlie associations between an exposure and an outcome. Within a quasi-experiment, the exposure of interest (eg, parent anxiety) is naturally occurring and not manipulated by the investigator. Unique design features are used to account for unmeasured variables that are confounded with the hypothesized causal environment, such as genetic relatedness between parents and offspring, to strengthen causal inferences.^34^^,^^35^ A range of genetically informed quasi-experimental research designs have been developed for this purpose, comparing family members for whom genetic relatedness is known or can be approximated.^36^ Family types integral to these designs include parents with adopted children, parents with children conceived via gamete or embryo donation, identical and nonidentical twin pairs with children, and parents with two or more children who are differentially exposed to the variable of interest. These designs help to tease apart the role of genetic and environmental transmission effects across generations. When combined with longitudinal data, they can also shed light on the direction of effects between generations.

### Genetically Informed Quasi-experiments

#### Adoption and In Vitro Fertilization Designs

When used for quasi-experimental purposes, adoption designs require data from parents and offspring adopted at birth.^37^ In such studies, similarities between birth parents and the adopted child reflect only genetic and prenatal influences because postnatal contact is absent (this means that passive gene–environment correlation cannot occur). Similarities between adoptive parents and their unrelated adopted child are free from confounding by genetic relatedness (again, passive gene–environment correlation cannot occur), so such studies are used to examine influence of the rearing environment on child development, independent of genetic effects. The in vitro fertilization (IVF) (or cross-fostering) design follows a similar premise for offspring conceived via gamete or embryo donation, who are essentially adopted at conception. In cases where embryos are implanted into unrelated mothers, the IVF design can be used to distinguish the influence of both prenatal and postnatal rearing environments from genetic effects.^38^ Longitudinal data using both adoption-at-birth and adoption-at-conception study designs allow for examination of environmentally mediated effects between generations, including parent-to-child and child-to-parent effects (eg, ^39^^,^^40^). Here, results can quantify the extent to which genetically influenced child traits evoke change in parent traits (referred to as evocative gene–environment correlation), which would increase parent–offspring trait associations.^27^ A major advantage of adoption designs is that they eliminate any effect of shared genes in associations between parents and offspring by design because they use genetically unrelated parent–offspring dyads. However, a caveat to this is that participants in adoption and IVF studies may not be representative of the general population, so results may not be generalizable, and sample sizes are usually small. Further, it is necessary to examine and control for adoption openness (ie, the degree of contact and knowledge between birth and adoptive families) and the possibility of selective placement (ie, when birth or donor parent characteristics are matched with adoptive parent characteristics), as these can violate the design assumptions and bias results by making adopted children more similar to their birth or donor parents.^41^

#### Children-of-Twins Designs

Children-of-twins designs require data on identical (monozygotic [MZ]) and fraternal (dizygotic [DZ]) adult twin pairs with children.^42^, ^43^, ^44^ In MZ twin families, offspring are just as genetically related to their parent’s identical twin, who is their aunt or uncle, as they are to their own parent (genetic correlation = .50 with both individuals). In DZ twin families, offspring are less genetically related to their aunt or uncle (genetic correlation = .25 on average) compared with their own parent (genetic correlation = .50). As such, if offspring are more similar to their aunt or uncle for any given trait in MZ compared with DZ families, an effect of shared genes is indicated. Following this logic, researchers statistically estimate and control for the role of shared genes between generations (ie, controlling for the effects of passive gene–environment correlation). Residual parent–child associations are unconfounded by genetic relatedness and comprise the environmental effect of parents on offspring and vice versa as well as any unmeasured confounding (ie, additional confounding that is not captured by controlling for shared genetic influences). Extensions of the design can include more than one child per parent and different combinations of adult siblings, including half-siblings and unrelated sibling-in-laws.^44^, ^45^, ^46^ It is also possible to model the influence of environments shared across all members of nuclear/extended families. Analyses are reliant on an equal environments assumption, positing that the children of MZ twins are not exposed to their parent’s twin any more so than the children of DZ twins (which has been found to hold true in previous research).^47^ Children-of-twins data have not yet been modeled longitudinally in research, and it is possible for the effects of evocative gene–environment correlation to inflate estimates of the parent’s causal influence on the child.

#### Sibling-Comparison Designs

Sibling-comparison designs are unique in that they do not rely on differentially related participants but instead on siblings who are differentially exposed to a given environment. Specifically, sibling-comparison designs require a sample of parents with at least 2 children, where sibling differences exist for the independent variable/exposure (eg, prenatal maternal anxiety).^48^ Siblings are naturally matched into family units where they broadly share many potential confounding variables, including their parents, home and family environment, and genetic factors (genetic correlation between siblings = .50 on average). Researchers compare groups of differentially exposed, family-matched siblings on an outcome of interest (eg, internalizing problems) to examine the exposure effect while eliminating within-family confounding. It can be assumed that genetic risk transmission is equal between siblings at a population level, on average, given the random nature of inheritance. As such, researchers simultaneously control for both unmeasured genetic and environmental confounding in families (this controls for more than just passive gene–environment correlation in families, as all aspects of the siblings’ shared environment is controlled for). Typically, in sibling comparison methodology, no distinction is drawn between parent-to-child and child-to-parent effects, and it is assumed that siblings do not significantly influence one another.^49^ Again, it is possible for the effects of evocative gene–environment correlation to inflate estimates of the parent’s causal influence on the child.

### Additional Sources of Confounding

The discussed quasi-experimental designs account for genetic confounding in different ways and require specific subpopulations of families on whom phenotypic data have been collected. These designs cannot control for all potential confounds, and each design is characterized by a different set of methodological caveats and assumptions. Across all designs, shared method variance can arise when parents report on both their own and their offspring’s symptoms, thereby inflating estimates of intergenerational associations in research. Further, the length of time elapsed between measurement of the exposure (parent anxiety) and outcome (child internalizing) can influence results, with concurrent associations typically being stronger than those with a lagged outcome (eg, ^50^^,^^51^). Results may also differ depending on child age or developmental period, participant sex, socioeconomic status, presence of comorbid diagnoses, or reliability of the measures used for data collection. As such, it is important that researchers consider both measured and unmeasured confounders while drawing on a range of quasi-experimental research designs and protocols to yield reliable and robust conclusions.^52^

### Aims

We conducted the first systematic literature review to our knowledge to identify all existing empirical research where authors have accounted for familial genetic confounding in associations between parent anxiety and offspring internalizing outcomes. We focused on quasi-experimental research. We excluded studies that involved experimental manipulation of anxiety state within families and observational research where controls were not included for unobserved sources of confounding. Results relating to prenatal and postnatal parent anxiety exposure were investigated separately, as they relate to distinct forms of anxiety exposure, with distinct hypothesized modes of transmission.^10^ We provided a narrative synthesis, critique, and meta-analysis of the retrieved literature to date. Our primary aims were to examine the following questions:

1. Is parent anxiety associated with offspring internalizing outcomes after accounting for familial genetic confounders?
2. If so, what can we tell about the direction of effects between parents and offspring?
3. If extracted data permit further analysis of moderator terms, to what extent is the magnitude of the parent–child association affected by methodological (eg, study design, reporter, time lag between exposure and outcome) and/or observed (eg, sex, age, socioeconomic status, comorbid parent depression, obstetric complications) covariates?

## Method

### Search Strategy

Our methods were registered in advance using PROSPERO; protocol number: CRD42019134977. Our search was conducted between July and September 2019 using Web of Science and Ovid (Embase, MEDLINE, Global Health, PsycINFO). The search was restricted to articles published in English. The following search terms were used to identify articles examining parent anxiety (note that parent terms and anxiety terms were combined to restrict the number of search results; see Supplement 1, available online, for full search strategy): mother∗ or matern∗ or father∗ or patern∗ or parent∗ or ∗natal AND anx∗ or phobi∗ or “social∗ anx∗” or “general∗ anx∗” or neurotic∗ or obsessive∗ or panic or agoraphobi∗. The following terms were included to identify articles that examined offspring outcomes and those that used a quasi-experimental design to control for potential genetic confounding (note that to ensure that we identified all possible internalizing outcomes examined to date, we did not restrict the search to predefined internalizing outcomes): child∗ or adolescen∗ or teen∗ or youth∗ or young or offspring or infan∗ AND twin or twins or sibling∗ or adoption or adopted or “in vitro fertilization” or “assisted conception” or “cross-fostering” or “instrumental variable” or “quasi-experiment∗” or causa∗ or genes or genetic or geno∗ or heritab∗.

The abstracts of all returned articles were screened independently by 2 authors (Y.I.A. and M.H.). Studies were excluded if there was clear evidence that criteria were not met, with agreement from both researchers. The reference lists of relevant review articles were screened to identify any articles that were missed from the search, and further searches were made to identify published articles from the authors of relevant conference abstracts. Full text screening for all retained studies was conducted by Y.I.A. and M.H., independently, to confirm eligibility. Disagreement was resolved through discussion with the senior author (T.A.M.). Data extraction from studies to be included in the meta-analysis was conducted by Y.I.A., checked by T.A.M. and another author (T.S.).

### Study Selection

Published studies presenting empirical research were included if they involved a population of human parents and offspring (no sex or age restrictions); examined associations between parent anxiety (measured at trait, symptom, or disorder level) and offspring internalizing outcomes (relating to withdrawal, somatic symptoms, anxiety, depression)^53^; and used a natural quasi-experimental research design to account for genetic relatedness in associations between parents and offspring (ie, intergenerational genetically informed research designs, which enable researchers to control for participant relatedness).^36^

Our search terms identified some studies of parental stress. We considered these studies to meet inclusion criteria if they exclusively measured feelings of stress (ie, anxiety symptoms), not stressful life circumstances. Further, our search terms identified publications using candidate gene approaches (using parent and child DNA) to control for the influence of specific shared genes in parent–offspring associations. Mental health phenotypes are typically classified as complex traits; ie, they are polygenic, influenced by hundreds of thousands of genetic variants across the genome, each exerting a very small effect.^54^, ^55^, ^56^ Therefore, studies accounting for the transmission of only a handful of genes (ie, candidate genes) between generations are insufficient to control for genetic confounding in parent–child associations for mental health phenotypes.^57^ The only genomic studies that thus met our inclusion criteria would be studies taking a genome-wide, polygenic approach to quantifying intergenerational genetic relatedness. Studies were excluded if they focused exclusively on populations with specific physical health problems (eg, cancer, seizures, low gestational age) or a diagnosed developmental disorder (ie, communication or learning disorders, motor disorders, attention-deficit/hyperactivity disorder, or autism spectrum disorders) or involved an experimental exposure or intervention.

**Figure 1:**
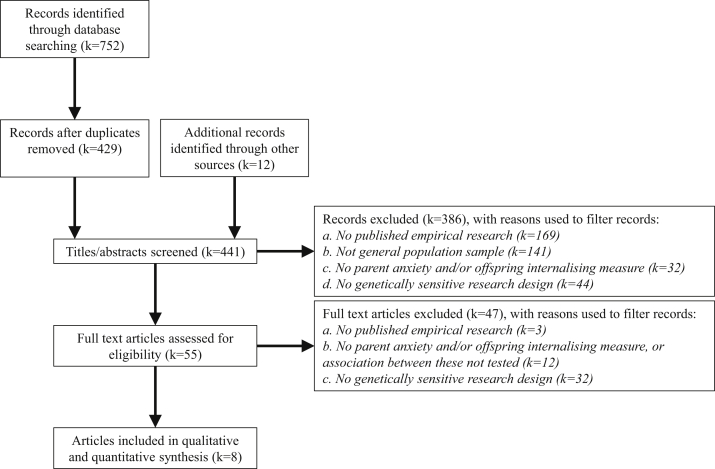
PRISMA Flowchart

Figure 1 presents the PRISMA flowchart. Of the 441 records screened, 8 publications met our inclusion criteria.^39^^,^^40^^,^^51^^,^^58^, ^59^, ^60^, ^61^, ^62^ Of the excluded publications, 7 included design features that accounted somewhat for bias by genetic confounding.^63^, ^64^, ^65^, ^66^, ^67^, ^68^, ^69^ These studies and our reasons for their exclusion are outlined in Supplement 2, available online. In brief, they were studies of prenatal anxiety exposure that used paternal anxiety symptoms as a negative control for environmentally mediated prenatal transmission and/or examined child exposure to parent state-level (ie, current, transitory) anxiety symptoms, while controlling for parent trait-level (ie, stable, longer-term) symptoms. We determined that these studies did not meet our criteria for a robust method to account for genetic relatedness in associations between parents and offspring.

### Data Extraction

Data relating to sample characteristics, measurement protocol and statistical analyses were extracted from each publication that met our inclusion criteria. All genetically informed effect estimates were initially extracted. Where authors published multiple effect sizes for the same set of variables (eg, where multiple analytic strategies were explored), we had to decide which estimates to include in the meta-analysis to derive a meaningful pooled result. For the 3 publications examining prenatal anxiety exposure, one examined both continuous and binary coded data.^59^ We selected results based on continuous scores, to be consistent with the 2 other publications.^51^^,^^58^ Two publications examining prenatal anxiety exposure reported separate effect estimates from analyses before and after adjusting for postnatal anxiety exposure,^58^^,^^59^ while the third reported only adjusted results.^51^ All effect estimates were retained to explore comparison of results that did vs did not attempt to isolate the effects of prenatal from postnatal exposure.

For the 6 publications of postnatal anxiety exposure, 2 reported both bivariate correlations and β estimates from structural equation models (ie, partial correlations) for the same set of variables, involving both cross-sectional and longitudinal data points.^39^^,^^40^ It was not possible to extract data from the structural equation models in an informative way for inclusion in the meta-analysis because saturated results were not presented in either publication (ie, some paths had been trimmed from the models). Of the bivariate correlation analyses, longitudinal correlations (ie, between parent anxiety exposure and future internalizing outcomes in offspring) were not informative on their own as prospective associations because they did not include correction for concurrent exposure to parental symptoms. As such, only the cross-sectional bivariate correlations were selected from these publications for inclusion in the meta-analysis. Cross-sectional effect estimates were available for inclusion in 3 of the 4 remaining publications examining postnatal anxiety exposure. For the one publication using only longitudinal data, the effect size with the shortest exposure-outcome time lag was selected (9 months), to conserve consistency in the meta-analysis.^61^ Two remaining longitudinal effect estimates (derived from 2 publications)^51^^,^^61^ were subsequently excluded, given that so few longitudinal estimates would be uninformative in the meta-analysis.

### Effect Size Calculations

Pearson’s correlation coefficient, *r*, was used as the uniform effect size across all studies, with CIs computed for each estimate using the R package compute.es.^70^ Pearson’s *r* is an appropriate effect size to use for associations between continuous variables, and results are easily interpretable.^71^ Nonindependent effect sizes derived from the same or overlapping samples in a single publication (eg, effect sizes at different child ages for families in a single cohort) were aggregated using the R package MAd (for meta-analysis with mean differences)^72^ to account for their correlation*.* Aggregation of correlated (ie, nonindependent) effect sizes within publications is required to prevent overestimation of the precision of the pooled effect size in meta-analysis, which occurs when findings based on the same data are incorrectly treated as unique.^73^^,^^74^ Aggregation of effect sizes within publications also prevents studies with more effect estimates from being given more weight in meta-analysis. Multilevel models are not appropriate to account for nonindependence of effect size estimates within a single publication.

Nonindependent effect sizes within each publication were aggregated first to create pooled effect sizes per publication (*r*_publication_). In sensitivity analyses, we aggregated nonindependent effect sizes within each cohort to create pooled effect sizes per cohort (*r*_cohort_) (see Figure S1, available online, for an example, depicting the aggregation of 14 effect sizes across 4 publications, all derived from one cohort). Aggregation of nonindependent effect sizes requires specification of their correlation. The magnitude of their correlation depends on the degree of overlap in the population sample, measures, and time points used for each estimate. As is typically the case in a meta-analysis, the correlations between dependent effect sizes within each publication and between publications were unknown, meaning that we had to specify a likely value. Given the potential for this specified correlation to impact results, we conducted 3 sensitivity analyses for each aggregation, testing results using a full range of possible correlations for the association between dependent effect sizes: *r* = .10, *r* = .50, and *r* = .90.

### Random-Effects Models

Meta-analytical models were conducted as multilevel random-effects models (REMs) using the R package metafor.^75^ Multilevel REMs allow for between-study heterogeneity and can be used to test for moderating effects when data permits (ie, methodological and observed covariates). First, a multilevel REM was used to pool Pearson’s *r* effect sizes from each publication (*r*_publication_) examining concurrent anxiety exposure. In this model, a source of variation was introduced for each cohort to account for random variance (ie, higher-order clustering) between cohorts and for each publication within each cohort. Next, in a sensitivity analysis, we conducted a standard REM to pool aggregated, independent Pearson’s *r* effect sizes from each cohort (*r*_cohort_). These results are not biased by nonindependent effect sizes, although they eliminate any information on the effects of moderating terms on the magnitude of associations within cohorts, relating to study design and sample characteristics in each publication.

Heterogeneity between effect sizes was assessed using the *I*^2^ statistic to examine whether study characteristics moderated the pooled effect size.^76^ The *I*^2^ statistic is the percentage of total variation in study estimates that is due to heterogeneity, or between-study variability (values <25% indicate low heterogeneity; 25%–75%, moderate heterogeneity; and >75%, high heterogeneity). Publication bias was evaluated visually using funnel plots, plotting effect sizes against their standard errors. Symmetrically distributed data points indicate absence of publication bias. The low number of included studies yielded insufficient statistical power to test for asymmetry using Egger’s linear regression.^77^

## Results

### Study Descriptions

The 8 retrieved papers were published originally between 2010 and 2019, using data derived from 4 independent cohorts located in northern Europe and America (N_total_ = 12,990).^39^^,^^40^^,^^51^^,^^58^, ^59^, ^60^, ^61^, ^62^ Each cohort had one quasi-experimental research design applied (adoption,^39^^,^^40^^,^^60^^,^^61^ IVF,^58^ children-of-twins,^62^ sibling-comparison^51^^,^^59^) and was mostly restricted to the study of one developmental period (infancy,^40^^,^^51^^,^^59^, ^60^, ^61^ middle childhood,^39^^,^^58^ or adolescence^62^) (Tables 1 and 2). The sibling-comparison sample (derived from the Norwegian Mother, Father and Child Birth Cohort Study [MoBa]) was far larger than all other samples combined. As shown in Tables 1 and 2, available information suggested that >90% of the rearing parents examined across publications were of European ancestry. No information was available for participant ancestry in the MoBa, although data from Statistics Norway suggest that approximately 90% of the Norwegian population had Norwegian-born parents and grandparents the year that MoBa recruitment ended (2009), indicating that MoBa participants would be predominantly of European ancestry.^78^ Ancestry data were ambiguous for participants in the children-of-twins sample (Twin and Offspring Study in Sweden [TOSS]; see footnote of Table 2), although again evidence suggests that participants were predominantly of European ancestry.^79^

**Table 1:** Studies of Prenatal Anxiety Exposure: Extraction of Quasi-experimental Data

Region	Cardiff-IVF cohort	MoBa cohort	
	United Kingdom	Norway	
Available information on participant ancestry	Offspring: 91.5% European	Information not available	
Quasi-experimental design	In vitro fertilization	Sibling-comparison	
Family unit, N	2 (1 parent, 1 child)	3 (1 parent, 2 children)	
**Reference**	**Rice*et al.*,2010**^**58**^	**Bekkhus*et al.*,2018**^**59**^	**Gjerde*et al.*,2020**^**51**^
Family units in quasi-experimental sample	205	5,935	11,553
Exposure period (gestational weeks)	Prenatal (31–40)	Prenatal (17–30)	Prenatal (30)
Exposure measure (reporter)	Anxiety/stress: 1 item completed retrospectively, 11-point response scale (self)	Anxiety: 5- and 8-item Hopkins Symptom Checklist, 4-point scale (self)	Anxiety: 8-item Hopkins Symptom Checklist, 4-point scale (self)
Parent relationship to child	Mother	Mother	Mother
Outcome period (child age, y)	Middle childhood (4–10)	Infancy (0.5, 3)	Infancy (1.5, 3, 5)
Outcome measure (reporter)	Anxiety: 6 items based on *DSM-IV*, 3-point response scale (mother)	Infant difficulties: 9-item Infant Characteristic Questionnaire, 7-point scale (mother) Emotional difficulties: 10-item Child Behavior Checklist, 3-point scale (mother)	Internalizing: 13-item Child Behavior Checklist, 3-point scale (mother)
Genetically informative analyses (estimates included in meta-analysis)^a^	(1) Multiple regression (standardized β = .21, longitudinal) (2) Multiple regression (standardized β = .11, longitudinal)	(1) Multiple regression (standardized β = .07, β = .02, longitudinal) (2) Multiple regression (standardized β = −.03, β = −.00, longitudinal)	Multilevel regression (standardized β = .01; longitudinal)^b^
Measured covariates considered in estimate extracted for meta-analyses^a^	(1) Child age, child sex, family social occupational class, antenatal complications (vaginal bleeding, admission to hospital for high blood pressure/edema, maternal cigarette smoking, maternal alcohol use, infant plurality) (2) Child age, child sex, family social occupational class, antenatal complications (vaginal bleeding, admission to hospital for high blood pressure/edema, maternal cigarette smoking, maternal alcohol use, infant plurality), maternal postnatal anxiety/depression	(1) None (2) Child sex, partner (dis)harmony, marital status, maternal education, antenatal complications (maternal prenatal cigarette smoking, maternal prenatal alcohol use, gestational age, birth complications, birth weight), somatic disease, maternal age, parity, maternal postnatal anxiety	Child age, child sex, parity, maternal postnatal anxiety/depression
Direction of effects assessed in publication	No	No	No

Note: Cardiff-IVF = Cardiff In Vitro Fertilisation study; MoBa = Norwegian Mother, Father and Childbirth Cohort Study

a Numbering indicates separate, genetically informative analyses, where authors did vs did not adjust for postnatal anxiety exposure.

b Regression coefficient standardized using the reported standard deviations (s) for the independent (x) and dependent (y) variables [zβ = β(sx/sy)].

**Table 2:** Studies of Postnatal Anxiety Exposure: Extraction of Quasi-experimental Data

Region	EGDS cohort I				TOSS cohort	MoBa cohort
	United States				Sweden	Norway
Available information on participant ancestry	Adoptive mothers, %: 91.4 European; 3.6 African; 2.5 Latino; 2.5 Other/Mixed. Adoptive fathers, %: 90.2 European; 5.0 African; 1.7 Latino; 3.1 Other/Mixed. Birth Mothers, %: 71.7 European; 11.4 African; 6.7 Latino; 10.8 Other/Mixed. Birth Fathers, %: 74.6 European; 8.7 African; 8.7 Latino; 8.0 Other/Mixed.				Participants: 100% European^a^	Information not available
Quasi-experimental design	Adoption				Children-of-twins	Sibling-comparison
Family unit, N	2 (1 parent, 1 child)				4 (2 parent, 2 children)	3 (1 parent, 2 children)
**Reference**	**Brooker*et al.*,2011**^**61**^	**Brooker*et al.*,2014**^**60**^	**Brooker*et al.*,2015**^**40**^	**Ahmadzadeh*et al.*,2019**^**39**^	**Eley*et al.*,2015**^**62**^	**Gjerde*et al.*,2020**^**51**^
Family units in quasi-experimental sample, N	361	361	349	305	871	11,553
Exposure period (child age, y)	Infancy (0.75)	Infancy (0.75)	Infancy (0.75, 1.5, .2.25)	Middle childhood (6, 7, 8)	Adolescence (11–22)	Infancy (0.5, 1.5, 3, 5)
Exposure measure (reporter)	Anxiety: 21-item Beck Anxiety Inventory, 4-point scale (self)	Anxiety: 21-item Beck Anxiety Inventory, 4-point scale (self)	Anxiety: 21-item Beck Anxiety Inventory, 4-point scale (self)	Anxiety: 20-item State-Trait Anxiety Inventory for Adults, 4-point scale (self)	Anxiety: 20-item Karolinska Scales of Personality, 4-point scale (self)	Anxiety: 8 item Hopkins Symptom Checklist, 4-point scale (self)
Parent relationship to child	Unspecified	Unspecified	Mother, father	Mother, father	Unspecified	Mother
Outcome period (child age, y)	Infancy (0.75)	Infancy (1.5, 2.25)	Infancy (0.75, 1.5, .2.25)	Middle childhood (6, 7, 8)	Adolescence (11–22)	Infancy (1.5, 3, 5)
Outcome measure (reporter)	Social inhibition: observational tasks (researcher)	Internalizing: 36-item Child Behavior Checklist, 3-point scale (mother, father)	Negative affect composite: 11-item Infant Characteristics Questionnaire, 7-point scale; 36-item Infant Behavior Questionnaire, 7-point scale; 19 item Toddler Behavior Assessment Questionnaire, 7-point scale (mother, father), observational tasks (researcher)	Anxiety: 13-item Child Behavior Checklist, 3-point scale (mother, father)	Anxiety: 7 items from Child Behavior Checklist, 3-point scale (mother, father); 7 items from Child Behavior Checklist, 3-point scale (self)	Internalizing: 13-item Child Behavior Checklist, 3-point scale (mother)
Genetically informative analyses (estimates included in meta-analysis)	Bivariate correlation (*r* = .00, cross-sectional)	Bivariate correlation (*r* = .23, longitudinal)	Bivariate correlation (*r* = .03, *r* = .02, *r* = .00, *r* = .19, *r* = .07, *r* = .08; cross-sectional)	Bivariate correlation (*r* = .16, *r* = .15, *r* = .20, *r* = .24, *r* = .11, *r* = 10; cross-sectional)	Children-of-twins structural equation model (standardized β = .25; cross-sectional)	Multilevel regression (standardized β = .05; cross-sectional)^b^
Measured covariates considered in estimate extracted for meta-analyses	None	None	None	None	Parent age, parent sex	Child age, child sex, parity, maternal depressive symptoms at each assessment (including prenatal), exposure and outcome at each time-point (including prenatal)
Direction of effects assessed in publication	No	No	Yes	Yes	No	No

Note: EGDS = Early Growth and Development Study; MoBa = Norwegian Mother, Father and Child Birth Cohort Study; TOSS = Twin and Offspring Study in Sweden.

a The data to support this statistic were ambiguous. In their article overviewing this cohort, Neiderhiser and Lichtenstein^79^ reported that participants were “in principle 100% Caucasian … consistent with the population of Sweden.”

b Regression coefficient standardized using the reported standard deviations (s) for the independent (x) and dependent (y) variables [zβ = β(sx/sy)].

Parent anxiety and offspring internalizing symptoms were measured along continuous scales in all analyses extracted for the meta-analyses. All publications used correlation coefficients and/or β estimates to evaluate intergenerational covariance (Tables 1 and 2). Parent anxiety was measured by self-report, using 5 different measures of adult anxiety across publications. Seven child internalizing constructs were assessed across the publications (eg, combined internalizing, negative affect, anxiety, social inhibition). Parents contributed at least partially to child symptom scores in all publications except one (where child social inhibition was measured solely by researcher observations).^61^ Results derived from the sibling-comparison or IVF datasets were subject to the greatest risk of shared method variance because only mothers’ reports were used to construct variables in these cohorts. Two publications (each using the same adoption sample at different developmental stages) examined the directionality of effects between generations and analyzed mother–child and father–child associations separately.^39^^,^^40^ A range of different measured covariates were accounted for across publications, each attenuating the crude parent–offspring correlation to varying degrees.

### Meta-analysis

#### Prenatal Anxiety Exposure

**Figure 2:**
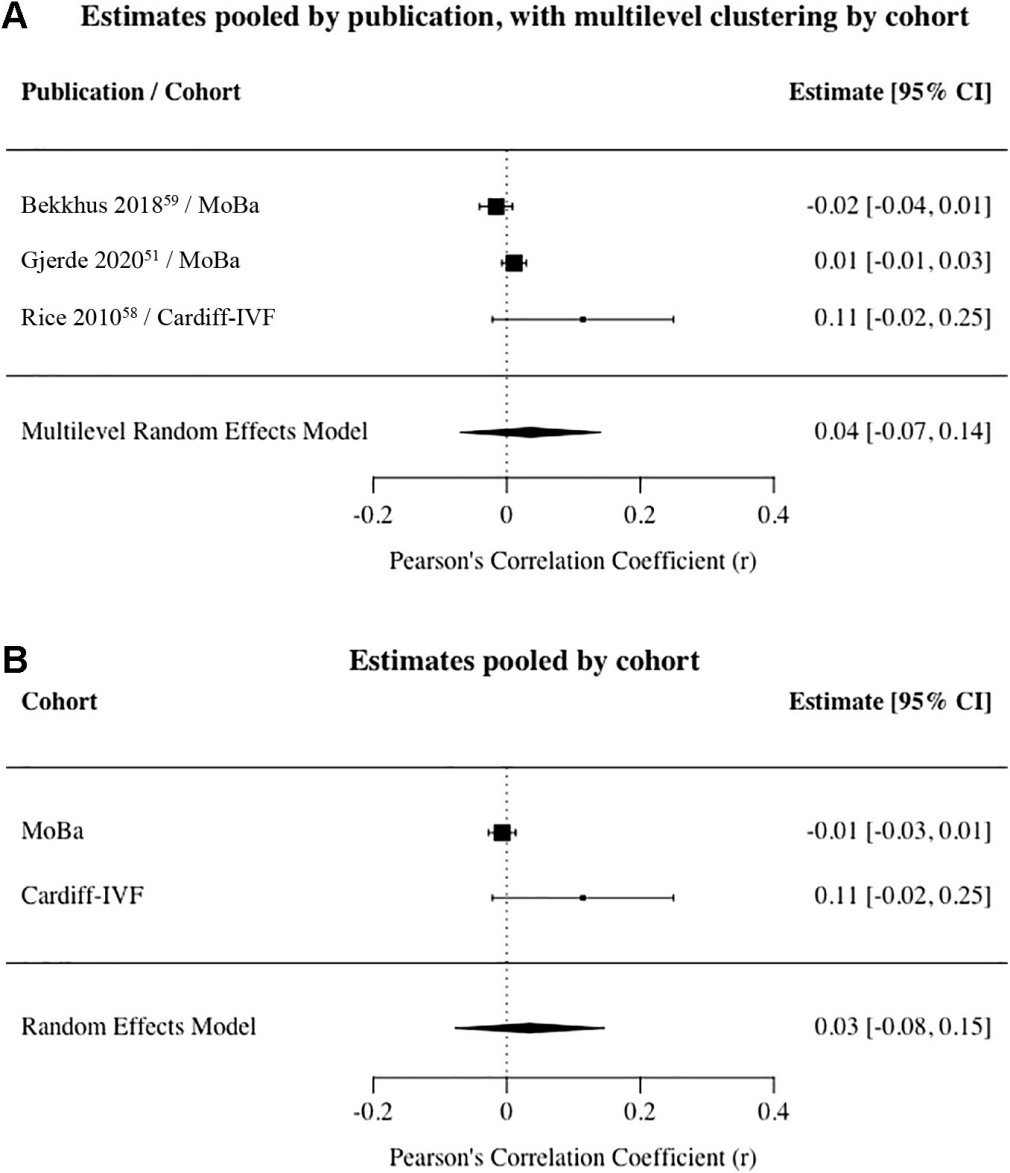
Association Between Prenatal Anxiety Exposure and Offspring Internalizing Outcomes ***Note:****(A) Estimates pooled by publication, with multilevel clustering by cohort. (B) Estimates pooled by cohort. Cardiff-IVF = Cardiff In Vitro Fertilisation study; MoBa = Norwegian Mother, Father and Child Cohort Study.*

Multilevel REM results showed a negligible and nonsignificant pooled effect size between prenatal anxiety exposure and infant internalizing outcomes using data from 3 publications (N_families_ >11,700; offspring age range, 0.5–10 years) that were corrected for genetic confounding and exposure to postnatal anxiety (*r* = .04; 95% CI: −.07, .14) (Figure 2A). Pooled estimates were equivalent in REM analyses using aggregated cohort data (Figure 2B). Two publications provided results that were unadjusted for postnatal anxiety exposure. REM analyses of these estimates revealed the pooled effect size to be larger than those using adjusted estimates, but still nonsignificant (*r* = .11; 95% CI: −.05, .28). Because there were only 3 publications examining prenatal anxiety exposure, statistical power was insufficient to test for heterogeneity of effect sizes.

#### Postnatal Anxiety Exposure

**Figure 3:**
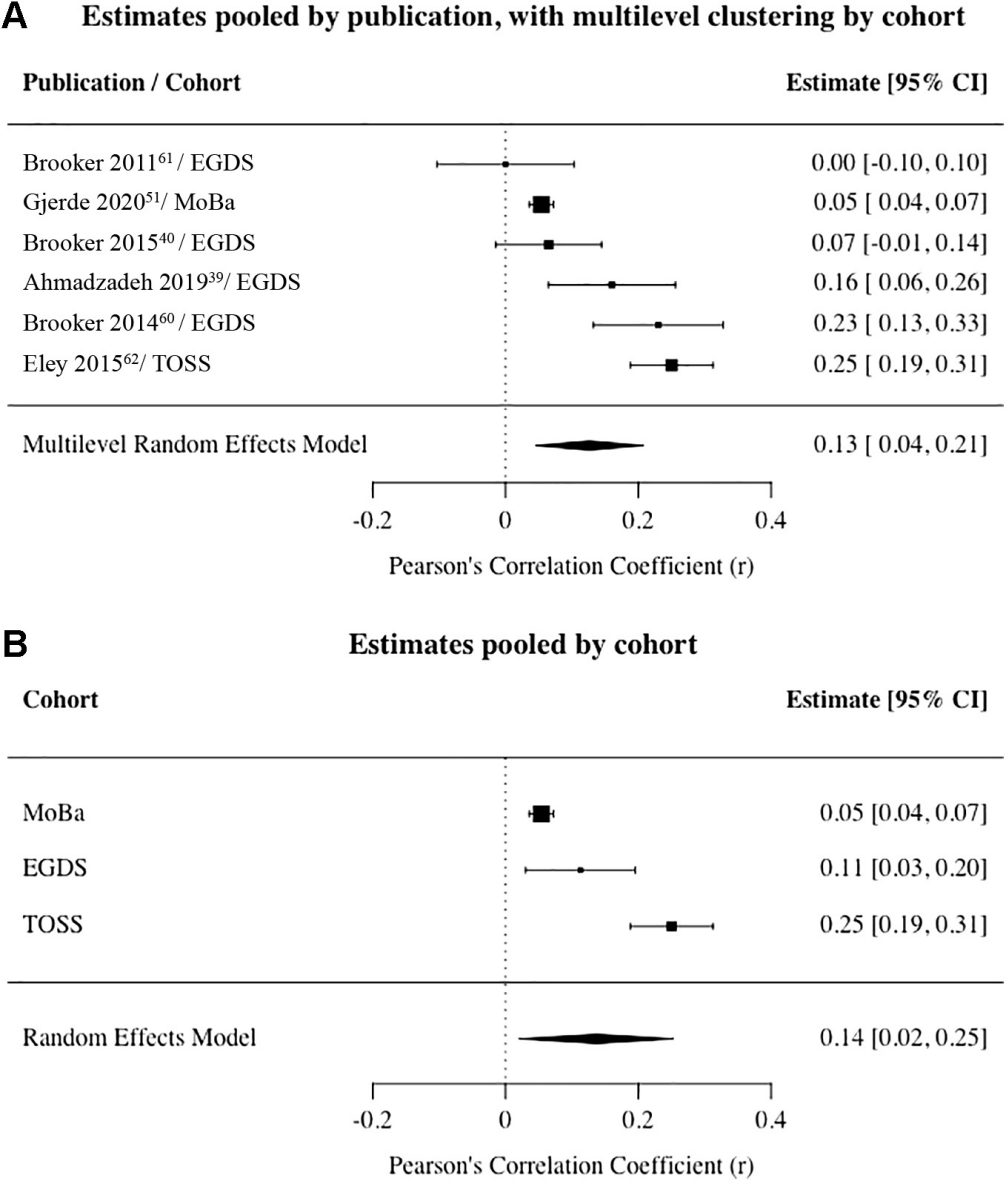
Association Between Postnatal Anxiety Exposure and Offspring Internalizing Outcomes ***Note:****(A) Estimates pooled by publication, with multilevel clustering by cohort. (B) Estimates pooled by cohort. EGDS = Early Growth and Development Study; MoBa = Norwegian Mother, Father and Child Cohort Study; TOSS = Twin Offspring Study in Sweden.*

Multilevel REM results showed a significant pooled effect size between concurrent anxiety exposure and offspring internalizing outcomes using data from 6 publications (N_families_ >12,700; offspring age range, 0.75–22 years) that were corrected for genetic confounding (*r* = .13; 95% CI: .04, .21) (Figure 3A). Results were comparable in analyses using effect sizes aggregated by cohort (Figure 3B). Results showed substantial levels of heterogeneity between publications (*I*^2^ = 90, suggesting that 90% of the χ^2^ statistic was explained by variation between studies of postnatal anxiety exposure). Assessment of relevant moderators to identify sources of heterogeneity was not feasible because the cohorts used were largely dissimilar in their sample and design characteristics. They could not be grouped and compared in meaningful ways, and statistical power would have been insufficient to explore variance explained by higher-order clustering (ie, multilevel REMs to examine moderation by covariates require meaningful variance between covariates).^80^ Of note, most publications used an adoption design, meaning that authors could not report estimates that were free from adjustment by genetic confounds (ie, all adoption results are adjusted by design for genetic relatedness because parents and offspring are not genetically related). Therefore, we were unable to compare effect sizes across levels of adjustment (ie, adjusted vs unadjusted for genetic confounds).

All presented models used aggregate effect sizes within publications/cohorts assuming a median correlation of *r* = .50, as suggested elsewhere.^72^, ^73^, ^74^ Results were consistent across the 3 sensitivity analyses run for each effect size aggregation (*r* = .10, *r* = .50, *r* = .90, by publication and by cohort) (see Figure S2, available online).

### Additional Observations

In the publications examining prenatal anxiety exposure, only the IVF design yielded a significant, nongenetic association, for offspring anxiety in middle childhood (standardized β = .21).^58^ However, this effect was attenuated and no longer significant after postnatal anxiety exposure was controlled for (standardized β = .11). In the 2 publications using sibling-comparison designs, researchers found no significant associations in any of their reported analyses with offspring during early childhood (standardized β range, −.03 to .07).^51^^,^^59^

As shown in Table 2, all effect sizes involving postnatal anxiety exposure were weak (standardized β and *r* range, .00 to .25). However, structural equation models in 2 publications using adoption designs showed that parent and child symptoms could prospectively predict one another across time, highlighting intergenerational, nongenetic, transactional effects during early and middle childhood.^39^^,^^40^ Results from these publications showed differences for mother–child vs father–child effects. For example, stronger evidence for an effect of a child’s symptoms on a father’s anxiety compared with a mother’s anxiety was observed during infancy,^40^ while an effect of a child’s symptoms during middle childhood was observed only for a mother’s anxiety and not a father’s anxiety.^39^ The only publication using a sibling-comparison design for postnatal analyses showed that mothers’ symptoms did not prospectively predict offspring internalizing symptoms, after controlling for genetic relatedness.

### Publication Bias

Studies with significant findings are more likely to be published in scientific journals, which increases risk of incorrect conclusions from systematic reviews of published literature and risk of false-positive or false-negative findings in meta-analytic results.^81^ For example, nonsignificant intergenerational associations are unlikely to be published, meaning that parents may appear more similar to their offspring when judging by the published literature alone. Funnel plots for our data are shown in Figure S3, available online, providing preliminary, albeit nonsignificant, evidence for publication bias. Only one publication reported null findings for any association between parent anxiety and offspring internalizing; however, the main focus of that study was on other phenotypes not relevant to this review, for which they had significant findings.^61^

## Discussion

Following a systematic literature search, we found only 8 publications where authors used a quasi-experimental research design to control for genetic confounding in associations between parent anxiety exposure and offspring internalizing outcomes. These publications used data from 4 independent cohort studies located in northern Europe or America, where each cohort had a different quasi-experimental research design applied. Low homogeneity between publications from different cohorts yielded low statistical power to test for moderation by methodological (eg, study design) or observed (eg, child age) covariates. Results highlight a striking need for new research, without which we remain ill-equipped to identify the pathways underpinning why parent anxiety symptoms are associated with the development of offspring internalizing problems.

### Mother’s Prenatal Anxiety Symptoms Were Not Associated With Offspring Internalizing Symptoms After Controlling for Genetic Relatedness

Results from 3 publications using data from 2 cohorts indicated that prenatal exposure to maternal anxiety is not associated with offspring internalizing symptoms via nongenetic mechanisms. Quasi-experimental research examining prenatal depression symptoms showed similar findings (also derived from the MoBa cohort evaluated in the present study).^82^ As such, quasi-experimental findings to date contradict existing literature on fetal programming in the context of familial risk for internalizing problems, which has been derived mostly from observational and/or animal studies (eg, 10, 11, 12, 13). We emphasize the need for new genetically informed investigations to produce a robust evidence base, looking across child development and into adulthood, including data from more diverse samples. Until new research is available, we encourage researchers and clinicians to consider the importance of genetic transmission and postnatal exposure in their work on maternal anxiety during pregnancy.

### Concurrent Associations Between Parent Anxiety and Offspring Internalizing Symptoms Remained Significant After Controlling for Genetic Relatedness

A small but significant association was found for concurrent anxiety exposure and child internalizing symptoms in quasi-experimental studies that accounted for parent–child genetic relatedness. This finding is consistent with a causal interpretation, potentially reflecting at least some direct, environmentally mediated influence between parents and offspring. However, this result is limited to cross-sectional data. It cannot inform on the direction of effects between parents and offspring or on the stability of associations across time. Meta-analyses of concurrent vs longitudinal associations were not feasible given the scarcity of available data.

Mixed findings were reported in the few publications that did include longitudinal analyses within their quasi-experimental design. Adoption data showed evidence consistent with parent anxiety predicting child internalizing symptoms within 2-year periods during early and middle childhood.^39^^,^^40^ The same data also showed evidence for child-to-parent effects, mirroring results from longitudinal studies that do not control for genetic relatedness between parent and child.^14^, ^15^, ^16^ However, researchers using a sibling-comparison design found that mothers’ postnatal anxiety symptoms did not prospectively predict offspring internalizing symptoms within a 5-year period.^51^ It is clear that further research is needed. Although we did not restrict our search by offspring age, we found only publications conducted during childhood. Genetically informed research on familial depression in Sweden suggests maintenance of parent–offspring associations into adulthood.^83^ It is unknown whether the same pattern holds for anxiety.

In sum, the data retrieved in our systematic search provide some evidence for nongenetic pathways between parent anxiety and concurrent offspring internalizing symptoms during childhood; however, longitudinal research is lacking, and so the direction of effects between generations remains unclear. This is an important message for clinicians working with parents experiencing anxiety symptoms: we currently cannot tell with confidence whether parents’ symptoms exert palpable, lasting influence on offspring internalizing outcomes. Furthermore, research has been limited to very homogeneous participant groups, and information is lacking as to the generalizability of results across populations.

### Considering the Role of Methodological Confounding

#### Bias by Quasi-experimental Design

More research is required before we can test the extent to which effect estimates were biased by each quasi-experimental design used. The largest parent–offspring association that we found was derived from the only publication to examine adolescent offspring—also the only publication to use a children-of-twins design.^62^ We cannot tell whether this reflects influence of the developmental period, research design, and/or other factors. In children-of-twins research, the influence of genetic relatedness on a parent–offspring correlation will be underestimated if statistical power is low, thereby inflating the unconfounded residual estimate.^44^^,^^84^ As such, statistical power issues could explain the relatively large effect size derived from the only children-of-twins publication.^62^ Conversely, the role of genetic relatedness in families can be overestimated in sibling-comparison research, thereby deflating the adjusted estimate. This is because confounding by genetic and environmental family factors is simultaneously corrected for, while assuming that symptoms in the exposed sibling do not influence symptoms in the nonexposed sibling.^48^^,^^49^ This could explain the relatively small effect sizes reported in the 2 sibling-comparison publications we included in meta-analyses.^51^^,^^59^ It is also possible for both children-of-twins and sibling-comparison designs to overcorrect for genetic relatedness if genetic factors comprise an integral part of the causal pathway in parent-to-child environmental transmission, rather than acting as confounders across generations.^85^ Further, in both designs, it is possible for the effects of evocative gene–environment correlation to inflate estimates of the parent’s causal influence on the child.

The limitations associated with statistically controlling for genetic effects are bypassed in adoption and IVF designs, where parents and offspring are not genetically related. These designs make it easier to distinguish parent-to-child and child-to-parent causal effects. However, they come at the cost of smaller and potentially less representative samples. For example, both parents of adopted and donor-conceived offspring go to great lengths to have a child, which may lead to differences in parent–offspring relationships compared with families raising naturally conceived, biological offspring.^86^ Further, adoptive parents typically have higher socioeconomic status compared with the birth parents of adopted children and with nonadoptive parents.^41^ Mothers in IVF samples may be older and experience higher levels of antenatal risk.^38^ Children adopted at birth are at higher risk for having experienced prenatal adversity and inheriting genes associated with psychopathology, and the experience of a child being raised by parents to whom he or she is not genetically related (as a result of adoption or donor conception) may also influence child development.^87^^,^^88^ As such, conducting new research using a range of quasi-experimental designs should help to balance the strengths and limitations of each, yielding more reliable and robust conclusions.^52^

#### Measurement Bias

It is likely that measurement bias accounts at least partially for the heterogeneity observed across our reported effect estimates. When working with the large samples required for genetically informative quasi-experiments, it can be methodologically and/or logistically impractical to include lengthy assessments and more than one reporter per family. For example, prenatal symptoms in the IVF study were reported by mothers using a single item, several years after pregnancy, alongside mothers’ reports of offspring internalizing.^58^ Recall bias and shared method variance may have inflated the parent–offspring correlation in this sample. In the only publication to eliminate risk of shared method variance, parents’ self-reports of anxiety were not associated with child symptoms (measured by researcher observations).^61^ However, researcher observations of young offspring in artificial situations may not have been as reliable as parents’ reports. Indeed, data from multiple reporters do not always converge. For example, in the adoption cohort, we saw low agreement between parent reports of offspring anxiety, with father–child anxiety associations observed only when using fathers’ reports for both child and self.^39^ When new research becomes available, it will be informative to test for moderation by aspects of publication measurement protocol to investigate influence on pooled results in multilevel REM analyses. In the meantime, it will be important for researchers to consider the perspectives of multiple reporters where possible and maintain clarity as to the potential impact of measurement bias on results.

#### Use of Observed Covariates

In each publication, the nature, number, and combination of observed covariates influenced the strength and meaning of the results. Authors attempted to correct their analyses in a range of ways across publications (eg, regressing out the effects of age, sex, and socioeconomic status) (Tables 1 and 2). Some effect estimates included in our meta-analyses included no correction for measured covariates and were arguably undercorrected (eg, adoption results that did not include correction for perinatal complications). In contrast, analyses in one publication using a sibling-comparison design involved use of several covariates.^51^ When our meta-analysis of concurrent anxiety exposure was computed without results from the sibling-comparison analyses (which comprised by far the biggest sample), it was reassuring to find that the pooled effect estimate increased only by .03 (see Supplement 3, available online). Further, controls for anxiety exposure at different developmental stages requires consideration. In the case of chronic parental anxiety, colinearity becomes an issue for statistically differentiating exposure effects at different periods (eg, prenatal vs postnatal anxiety effects). That is, if anxiety symptoms before and after the child’s birth are highly correlated, controlling for variance in one period will remove variance in the other. This could explain why the prenatal anxiety association in the IVF study became nonsignificant after controlling for postnatal anxiety exposure (although analyses of postnatal exposure that included correction for prenatal symptoms did not find the same phenomenon, as residual postnatal symptoms remained predictive of offspring internalizing).^39^^,^^40^^,^^51^^,^^58^ Going forward, we encourage researchers to report both unadjusted and adjusted results, as Bekkhus *et al*.^59^ did, alongside information on the variance explained by each covariate to help in future research efforts to combine results.

### Further Avenues for Research

#### Expanding Analyses Beyond Parent–Offspring Dyads

The majority of research used in this review is focused on mother–child dyads. Where possible, it will be informative to take a more holistic approach to intergenerational research, considering fathers, siblings, and extended family members as well as the myriad social, economic, and societal factors that can influence participants’ mental health. For example, modeling both mother–child and father–child associations concurrently across time shows transactional influences between all individuals.^39^ Going forward, researchers could also include sibling effects in research and avoid the bias associated with selecting only one child per family for analyses (or 2 differentially exposed siblings).^89^ This could be possible in the Early Growth and Development Study (EGDS), where data are now collected on both birth and adoptive siblings.^37^ Information on multiple children per parent is also available in MoBa, where siblings can be included in multiple-children-of-twins models.^44^ These can be used to examine moderation by environments shared within families (eg, family composition and social support) and between families (eg, cultural and societal factors)^44^ while also including data on 2 parents to address issues surrounding assortative mating.^45^ The consequences of parents’ resemblance in anxiety has not yet been considered in genetically informed, intergenerational research. In sum, researchers should strive to move beyond analyses of only mother–child dyads to ensure validity and generalizability of results across families.

#### Cohorts That Were Not Designed for Quantitative Genetic Research

The quasi-experimental designs used in this review require highly specific, large-scale family samples. That we identified only 8 publications using data from only 4 cohorts is telling of the challenges associated with collecting these data. Several publications that we excluded in our systematic search used data from large-scale population studies (eg, Generation R and the Avon Longitudinal Study of Parents and Offspring) that are rich in phenotypic information but lacking the targeted recruitment required for traditional, pedigree-based genetic research (eg, adoptive parents or twins with children).^63^, ^64^, ^65^^,^^67^^,^^68^ Rapidly evolving methods in genomic research may soon provide novel opportunities for these cohorts, using participant DNA to examine intergenerational genetic transmission.

At the present time, genomic research for complex traits remains limited by a ceiling effect, whereby results reflect only the additive effects of genetic variants tagged on DNA arrays, excluding nonadditive effects or rare variants.^90^ Until this is addressed, genomic methods cannot adequately control for genetic relatedness when examining associations between parent and child traits in a way that is comparable with the control achieved in adoption, sibling-comparison, or children-of-twins research. When whole-genome methods become possible, analyses can involve use of polygenic scores in parents and offspring to examine the role of transmitted vs nontransmitted genetic variants in phenotypic associations across generations (eg, 91, 92, 93). The principles of Mendelian randomization can also be used to examine environmentally mediated, parent-to-child causal pathways, using parent genes as instrumental variables.^94^ Further, genomic variance decomposition methods can be used to partition the influence of parent and offspring genetic influence on traits when genome-wide single nucleotide polymorphism data have been collected from family members (eg, using M-GCTA, Trio-GCTA, or relatedness disequilibrium regression).^95^, ^96^, ^97^ We may soon be able to decompose covariance in traits across generations using estimates of single nucleotide polymorphism–based heritability. With rapid advances in genomic research, we may be on the brink of a new era for advancing our understanding of familial risk for anxiety and internalizing.

Some limitations of our methodology require emphasis. To pool together all available data, we combined a mix of bivariate and partial correlations. This limited our ability to directly compare estimates between publications, where different adjustments were made for observed covariates. We did not distinguish different types of internalizing problems among offspring, but instead pooled available data relating to child anxiety, negative affect, social inhibition, and other emotional difficulties. We cannot tell whether findings would differ by child disorder subtype. This results from lack of available data, meaning we could not test for moderating terms in multilevel REM analyses. All reviewed publications included a quasi-experimental design to account for genetic transmission effects in parent–offspring associations (ie, controlling for passive gene–environment correlation), and in our discussion of their findings we consider the possibility of child-to-parent evocative effects. However, we do not consider the possible action of gene-by-environment interaction in families, whereby genetic effects on traits vary in relation to individuals’ contexts or environments and vice versa.^98^ Gene-by-environment interactions are not modeled in any of the publications that we review and thus represent an important avenue for future research in the context of exposure to parental anxiety. Finally, all data used in our meta-analysis were derived from participants located in northern Europe or America. Ancestry data suggested that participants were predominantly of European descent. We highlight the need for new research in more representative samples in terms of geographical regions and participant ancestry. Without efforts to improve diversity in research participation, we risk preserving a cycle of scientific evidence, and subsequent evidence-based policy, based on groups who are, on average, privileged members of society.^99^

### Summary

Quasi-experimental designs can help to control for the effect of genetic relatedness in similarities between parents and offspring. We sought to investigate whether associations between parent anxiety symptoms and offspring internalizing symptoms can be explained via nongenetic mechanisms. We found the existing literature to be limited, with only 8 genetically informed studies published, using data from only 4 cohorts. In a meta-analysis of the available data, we found no evidence to suggest that maternal prenatal anxiety symptoms exert influence on the development of offspring symptoms via nongenetic mechanisms. However, we show that during childhood parent anxiety symptoms are associated with concurrent internalizing symptoms in offspring via nongenetic mechanisms.
