# Supplementary material for: Systematic Review and Meta-analysis of Genetically Informed Research: Associations Between Parent Anxiety and Offspring Internalizing Problems
Source: J Am Acad Child Adolesc Psychiatry. 2021 Jul;60(7):823–40. doi: 10.1016/j.jaac.2020.12.037 (PMC8259118; doi:10.1016/j.jaac.2020.12.037)
Supplement: Supplementary Material [file mmc1.docx]

**Ahmadzadeh, Y. I., Schoeler, T., Han, M., Pingault, J.-B., Creswell, C., & McAdams, T. A. (2021). Systematic Review and Meta-analysis of Genetically Informed Research: Associations Between Parent Anxiety and Offspring Internalizing Problems. *Journal of the American Academy of Child & Adolescent Psychiatry*.**

**Supplement 1. Full search strategy**

"mother* anx*" OR "matern* anx*" OR "father* anx*" OR "patern* anx*" OR "parent* anx*" OR "*natal anx*"

OR "mother* phobi*" OR "matern* phobi*" OR "father* phobi*" OR "patern* phobi*" OR "parent* phobi*" OR "*natal phobi*"

OR "mother* social* anx*" OR "matern* social* anx*" OR "father* social* anx*" OR "patern* social* anx*" OR "parent* social* anx*" OR "*natal social* anx*"

OR "mother* general* anx*" OR "matern* general* anx*" OR "father* general* anx*" OR "patern* general* anx*" OR "parent* general* anx*" OR "*natal general* anx*"

OR "mother* neurotic*" OR "matern* neurotic*" OR "father* neurotic*" OR "patern* neurotic*" OR "parent* neurotic*" OR "*natal neurotic*"

OR "mother* obsessive*" OR "matern* obsessive*" OR "father* obsessive*" OR "patern* obsessive*" OR "parent* obsessive*" OR "*natal obsessive*"

OR "mother* panic" OR "matern* panic" OR "father* panic" OR "patern* panic" OR "parent* panic" OR "*natal panic"

OR "mother* agoraphobi*" OR "matern* agoraphobi*" OR "father* agoraphobi*" OR "patern* agoraphobi*" OR "parent* agoraphobi*" OR "*natal agoraphobi*"

AND

child* OR adolescen* OR teen* OR youth* OR young OR offspring OR infan*

AND

twin OR twins OR sibling* OR adoption OR adopted OR "in vitro fertilization" OR "assisted conception" OR "cross-fostering" OR "instrumental variable" OR "quasi-experiment*" OR causa*

**Supplement 2. Discussion of seven excluded publications that accounted somewhat for bias by genetic confounding, but were not classified as using robust, genetically sensitive quasi-experiments**

Of the 441 records screened, only eight publications met inclusion criteria. Of the excluded publications, seven included design features that accounted somewhat for bias by genetic confounding. These studies and our reasons for their exclusion are outlined below:

Two studies of exposure to prenatal maternal anxiety used paternal anxiety symptoms as a ‘negative control’ for environmentally mediated prenatal transmission.^1,2^ The logic behind this design is that children can be exposed to *maternal* prenatal anxiety but are not directly exposed to *paternal* prenatal anxiety. Thus, equivalent prediction of child internalising by maternal and paternal prenatal mood would support a role for genetic transmission and provide evidence against an effect of foetal exposure to maternal anxiety. In the first of these studies, measures of paternal pre- and postnatal mood were included as covariates in analyses of mother-child associations, showing that mothers’ prenatal anxiety persistently predicted offspring outcomes across childhood.^1^ In the second publication, authors directly compared mother-child and father-child associations, demonstrating that only maternal prenatal anxiety symptoms predicted internalising problems in adolescent offspring.^2^ While these studies do go a long way towards controlling for potential genetic confounding between prenatal maternal anxiety and child internalising, the authors were unable to control for differences in offspring exposure to maternal and paternal anxiety symptoms *postnatally*. If offspring spent more time with mothers compared to fathers postnatally (which is likely in the societies they focus on), results could be skewed towards stronger mother-child associations. As such, we decided that these designs did not match our inclusion criteria. Next were studies that examined child exposure to parent state-level (i.e., current, transitory) anxiety symptoms, while controlling for parent trait-level (i.e., stable, longer-term) symptoms.^1,3-7^ Although not presented as such in these publications, measures of parent trait-level symptoms could be considered a proxy for overall genetic risk. However, any residual association involving the state-level symptoms could still be attributable to genetic mechanisms, so we decided to exclude publications using this design.

**References**

1. O'Donnell KJ, Glover V, Barker ED, O'Connor TG. The persisting effect of maternal mood in pregnancy on childhood psychopathology. *Dev Psychopathol.* 2014;26(2):393-403.

2. Capron LE, Glover V, Pearson RM, et al. Associations of maternal and paternal antenatal mood with offspring anxiety disorder at age 18 years. *J Affect Disord.* 2015;187:20-26.

3. Henrichs J, Schenk JJ, Schmidt HG, et al. Maternal pre- and postnatal anxiety and infant temperament. The generation R study. *Infant and Child Development.* 2009;18(6):556-572.

4. Aktar E, Majdandzic M, de Vente W, Bogels SM. The interplay between expressed parental anxiety and infant behavioural inhibition predicts infant avoidance in a social referencing paradigm. *Journal of Child Psychology and Psychiatry.* 2013;54(2):144-156.

5. O'Connor TG, Heron J, Golding J, Beveridge M, Glover V. Maternal antenatal anxiety and children's behavioural/emotional problems at 4 years. Report from the Avon Longitudinal Study of Parents and Children. *The British journal of psychiatry : the journal of mental science.* 2002;180:502-508.

6. O'Connor TG, Heron J, Golding J, Glover V. Maternal antenatal anxiety and behavioural/emotional problems in children: a test of a programming hypothesis. *Journal of child psychology and psychiatry, and allied disciplines.* 2003;44(7):1025-1036.

7. Van den Bergh BR, Marcoen A. High antenatal maternal anxiety is related to ADHD symptoms, externalizing problems, and anxiety in 8- and 9-year-olds. *Child Dev.* 2004;75(4):1085-1097.

**Supplement 3. Re-running the meta-analyses for postnatal exposure to parental anxiety, excluding estimates derived from the sibling-comparison design**

The effect estimate for postnatal anxiety exposure in the MoBa was unique in being derived from a sibling comparison design.^1^ The sibling-comparison design corrects for all unmeasured covariates shared between siblings in a family. This includes social factors as well as the parental genome, while the other quasi-experimental designs included in our meta-analysis account only for genetic factors. As such, the MoBa estimate included more controls compared to those from other cohorts, which may have attenuated the parent-offspring correlation to a greater degree. When excluding the MoBa data from the REM of postnatal anxiety exposure, meta-analytic results revealed a significant pooled correlation of *r*=.16 (90% CI .03, .29), compared to *r*=.13 (90% CI .04, .21).

**References**

1. Gjerde LC, Eilertsen EM, Eley TC, et al. Maternal Perinatal and Concurrent Anxiety and Mental Health Problems in Early Childhood: A Sibling-Comparison Study. Child Development. 2018;0(0).

**Figure S1. Non-independent effect sizes derived from the Early Growth and Development Study (EGDS) cohort**

Figure shows the number of effect estimates (grey boxes) extracted from the EGDS adoption cohort. They are non-independent, derived from overlapping individuals and datapoints, nested within a single cohort. The magnitude of the correlation between effect estimates will vary depending on the degree of overlap in the sub-sample, measures and timepoints used. Effect estimates were aggregated within publications to account for their correlation *(r*_publication_)*.* In a more stringent approach, effect estimates were aggregated within cohorts to account for their correlation *(r*_cohort_).

**Figure S2. Meta-analytic findings in analyses using *r=*.10 and .90 to aggregate non-independent effects across publications and cohorts**

***Prenatal Anxiety Exposure***

**A. Estimates pooled by publication, with multilevel clustering by cohort
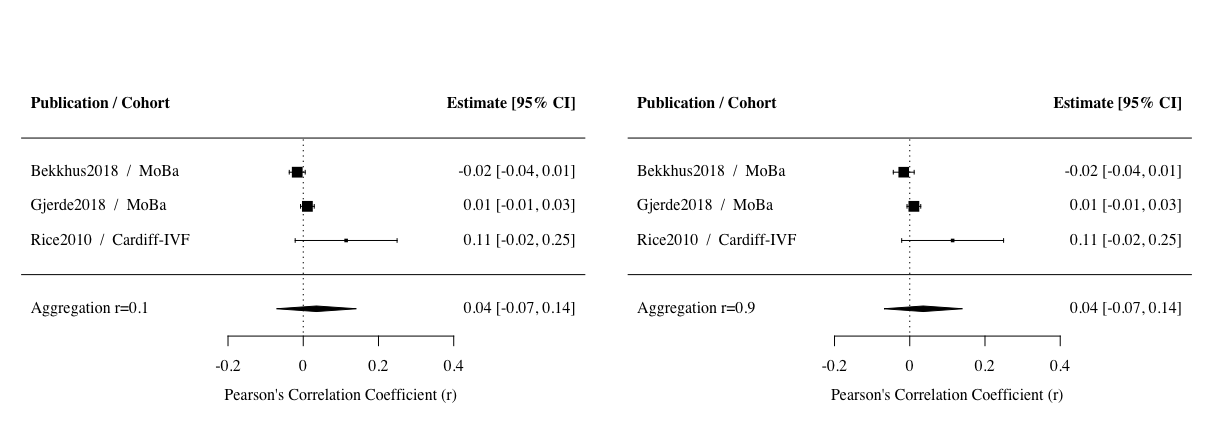
**

**B. Estimates pooled by cohort
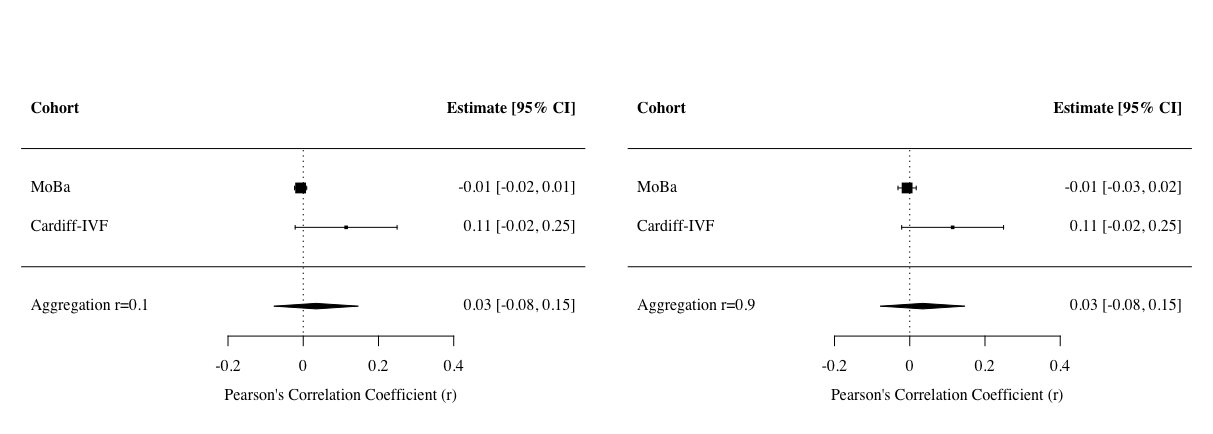
**

***Postnatal Anxiety Exposure***

**A. Estimates pooled by publication, with multilevel clustering by cohort**

**
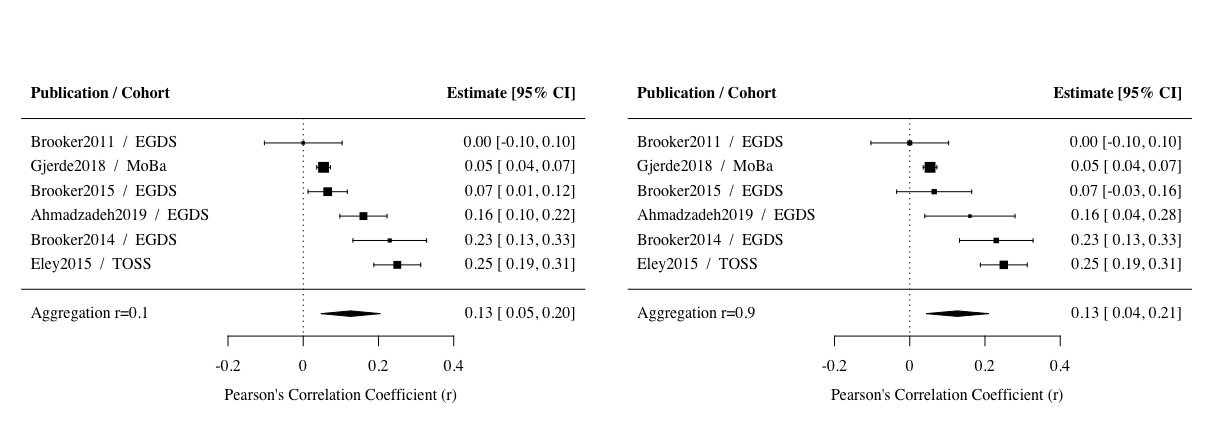
**

**B. Estimates pooled by cohort*
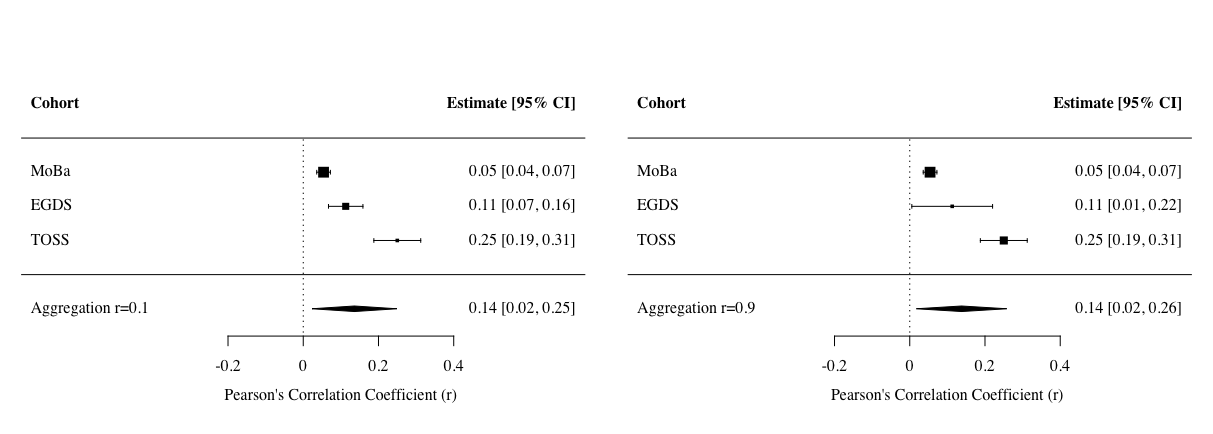
***

**Figure S3. Funnel plots for sampling variance by effect size Pearson’s *r***

**
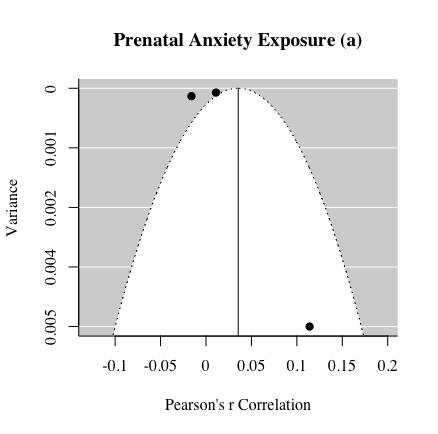

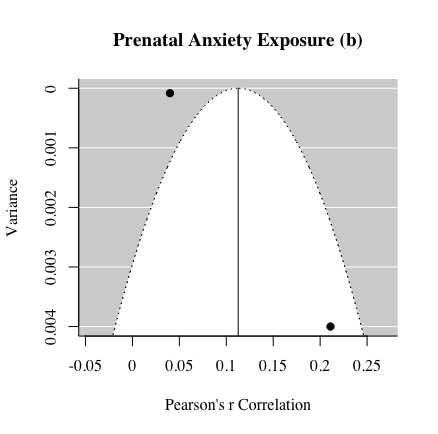

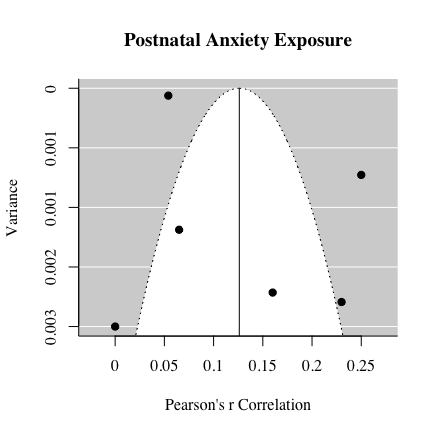
**

Funnel plots for sampling variance by effect size Pearson’s *r* to evaluate publication bias. The vertical solid line represents the pooled effect sizes Pearson’s *r* from the multilevel random effects models, the curved dotted lines show the expected 95% confidence intervals around the summary effect size. A funnel plot that is symmetrical with respect to the pooled effect size (vertical solid line) indicates that the effect size estimates differ between small- and large-sized studies. For prenatal anxiety exposure, a = estimates have been adjusted for postnatal anxiety exposure; b = estimates have not been adjusted for postnatal anxiety exposure. Some evidence for funnel plot asymmetry is suggested for in all panels, although robust conclusions cannot be drawn from so few studies. Egger’s regression test is underpowered to detect significant asymmetry.
